# Co-localization of areas of delayed mechanical activation and areas of myocardial scar

**DOI:** 10.1186/1532-429X-11-S1-O25

**Published:** 2009-01-28

**Authors:** Jana G Delfino, Brandon K Fornwalt, Calvin R Kurz, Jack A Talsma, John N Oshinski

**Affiliations:** 1 Emory University, Atlanta, GA USA; 2 Georgia Institute of Technology, Atlanta, GA USA; 3 Emory University/Georgia Institute of Technology, Atlanta, GA USA

**Keywords:** Cardiac Resynchronization Therapy, Short Axis Slice, Myocardial Scar, Ischemic Heart Failure, Delay Contrast Enhancement

## Objective

To determine the overlap between the regions of delayed mechanical activation and regions of myocardial scar tissue.

## Introduction

The greatest benefit from Cardiac Resynchronization Therapy (CRT) is likely achieved when the LV pacing lead is placed in the area with the greatest mechanical activation delay[1]. However, in patients with ischemic heart failure, the region with the greatest mechanical activation delay *may* correspond to an area of myocardial scar. Placing the pacing leads in areas of scar tissue will result in nonresponse to CRT[2]. The co-localization of areas of delayed mechanical activation and myocardial scar in patients with previous MI is not known.

## Methods

16 patients with a prior history of myocardial infarction (MI) were studied six-months post infarct. Cine SSFP images were obtained in the 2-chamber, 4-chamber, and short axis orientations. Following a double-dose injection (0.2 mmol/kg) of Gd-DPTA, delayed contrast enhancement (DCE) images were obtained at the same locations as the cine SSFP images.

Mechanical activation delays were calculated from the cine SSFP images. Radial displacement curves showing movement of the endocardial border toward the LV center of mass were calculated for 360 chords around each short axis slice. Displacement curves were averaged throughout the entire myocardium to generate a global displacement curve. Cross correlation analysis between the global curve and the curve for each chord was done to determine the mechanical activation delay. A cross correlation delay of 31 msec was used as the threshold to determine mechanical activation delay[3]. Mechanical activation delay information for the entire myocardium was mapped onto the standardized AHA Bullseye model.

Endocardial and epicardial borders were traced on the short axis DCE images. A separate region of interest was drawn around the area of infarct. Each slice of the myocardium was resampled at 360 chords around the heart, and each chord was assigned a value between 0 and 1 depending on the scar burden at that location (1 = transmural infarct, 0 = no infarct). The analysis was repeated for each short axis slice. DCE information for the entire myocardium was displayed on the standardized AHA Bullseye model of the myocardium.

Areas of overlap between mechanical activation greater than 31 msec and scar burden >50% transmurality were examined.

## Results

**Figure 1:**
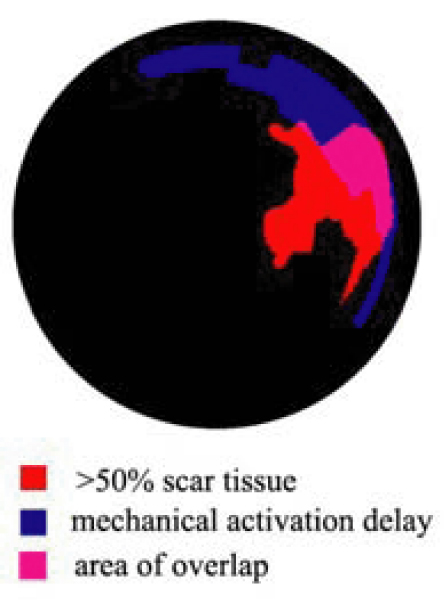
**An example patient showing regions of >50% scar tissue, regional mechanical activation delay, and regions of overlap**. In this example, 32% of the region of delayed mechanical activation was also a region of >50% scar tissue.

A scar burden of >50% was seen in 8.6 +/- 6.7% of the LV; mechanical activation delay greater than 31 msec was seen in 8.0 +/- 4.0% of the LV. On average, only 14.6 +/- 14.7% of the region of greatest mechanical activation delay was also an area of >50% scar tissue. However, this value varied greatly between patients and ranged from 0 to 40%. In the two patients where there was no overlap, scar burden was <5% of the myocardium. See Figure 1.

## Conclusion

The region of greatest mechanical activation delay does not necessarily correspond to the region of myocardial scar tissue.
